# Laparoscopic duodenum-preserving pancreatic head resection in 459 patients for precancerous, cystic neoplasms, and neuroendocrine tumors. Perioperative outcome: systematic review and meta-analysis

**DOI:** 10.1007/s00464-026-12585-z

**Published:** 2026-02-06

**Authors:** Hans G. Beger, Yang Yinmo, Benjamin Mayer, Bertram Poch

**Affiliations:** 1 University of Ulm, Albert-Einstein-Allee 23, 89081 Ulm, Germany; 2 Hepatobiliary and Pancreatic Surgery Department, Peking University First Hospital, Beijing, People’s Republic of China; 3 Institute for Epidemiology and Medical Biometry, Ulm University, Ulm, Germany; 4 Centre for Oncologic, Endocrine and Minimal Invasive Surgery, Donau-Klinikum Neu-Ulm, Neu-Ulm, Germany

**Keywords:** Pancreatic head resection, Laparoscopic duodenum-preserving pancreatic head resection, Parenchyma-sparing resection of cystic neoplasms, Neuroendocrine tumors, Laparoscopic pancreatic resection

## Abstract

**Background:**

With regard to laparoscopic approach, the objective arises whether standard multiorgan Whipple resection (PD) or parenchyma-sparing procedures (DPPHRt) are the most qualified surgical treatments for benign, premalignant neoplasms.

**Methods:**

Pubmed, Embase, Medline, and Cochrane Libraries were searched for studies reporting results and late outcomes after laparoscopic DPPHRt (L-DPPHRt) and laparoscopic PD (L-PD) for benign tumors. Data of 19 cohort studies including 459 patients were assessed. Results of six controlled trials comprising 129 L-DPPHRt and 205 L-PD for benign neoplasms were compared.

**Results:**

L-DPPHRt was performed for 123 IPMNs, 44 MCNs, 98 SPNs, 102 SCNs, and 59 PNETs. 90-day mortality was 2 of 459 patients (0.43%). Pancreatic fistula *B*/*C* occurred in 83 patients (18.08%) and biliary fistula in 35 patients (7.62%). Incidence of POPF *B* + *C* following complete and incomplete L-DPPHRt was 36/256 pats. (14.06%) and 40/167 pats. (23.95%) (*p* = 0.030), respectively. LHS was 14.24 days (mean). Laparoscopic total DPPHR unveiled very low risk of hospital mortality (1/459 pats.;0.21%), reoperation (9/364 pats.;2.47%), DGE (14/280 pats.;5.0%), CBD stenosis (2/459 pats.;0.43%), and ischemic lesion of CBD (2/459 pats.;0.43%). Comparing 129 L-DPPHRt with 205 L-PD patients revealed overall mean values of 239. vs. 343 min. for OP time and 128 ml vs. 240 ml for estimated blood loss. Meta analysis using standardized mean difference (SMD) demonstrated these differences to be significant (OP time: SMD − 1.20, 95% CI − 2.08 to 0.31; *p* = 0.008; blood loss: SMD − 1.77, 95% CI − 2.87 to − 0.66; *p* = 0.002). L-DPPHRt was associated with better intraoperative and early postoperative performance.

**Conclusions:**

Laparoscopic DPPHR for cystic neoplasms and PNETs is a low-risk procedure leading to cure of patients. L-DPPHRt accomplishes the most appropriate goals for treatment of patients with benign, premalignant, cystic neoplasms, and PNETs (> 2 cm) of the pancreatic head.

**Supplementary Information:**

The online version contains supplementary material available at 10.1007/s00464-026-12585-z.

The standard treatment for malignant tumors of the pancreatic head is Whipple operation or pylorus-preserving pancreaticoduodenectomy (PPPD). Due to improvements of surgical techniques, procedure standardization, multidisciplinary management of perioperative care, and expertise in many gastrointestinal surgical institutions, Whipple resection/PPPD (PD) is routinely used for benign, premalignant cystic neoplasm and pancreatic neuroendocrine tumor (PNET) [1]. With the development of minimally invasive laparoscopic technologies, Whipple resection and PPPD are increasingly replacing open PD for tumors of the pancreatic head. Laparoscopic abdominal procedures have the advantage of a reduced operative trauma and are associated with diminished intraoperative blood loss, faster early postoperative recovery, and shortening of postoperative hospital stay.

However, PD encompasses a multiorgan resection and, even with laparoscopic or robotic-assisted technique, PD is a complex and demanding procedure, associated with considerable risks for surgery-associated complications and significant hospital mortality. Whipple resection and, to a similar extent, PPPD are associated with a significant metabolic morbidity [2]. Pancreatic endocrine and exocrine insufficiency after PD is caused by duodenectomy and resection of the first jejunal loop, and to a lesser degree by resection of pancreatic head tissue [2, 3]. New onset of diabetes mellitus is observed in 12–20% of patients [4], preoperatively mild diabetes mellitus (DM) rendered postoperatively into insulin-dependent diabetes mellitus in up to 40% [5]. Pancreatic exocrine insufficiency leading to persisting diarrhea and steatorrhea is observed in 50–60% of patients, when stool fat content or stool elastase is measured [6, 7]. PD for benign tumors is associated with non-alcoholic fatty liver disease in 25–35% [8]. Non-alcoholic fatty liver disease (NAFLD) patients have a risk for development of steatohepatitis and steatocirrhosis in 5–8% [9]. Cholangitis or stenosis of the biliary-intestinal anastomosis is observed in 8–13% of patients after PD [9, 10], of them 3–5% suffer lifelong refractory attacks of cholangitis [11]. The frequency of early and late postoperative complications and the risk of long-term metabolic morbidity have limited the use of PD for benign tumors and premalignant cystic neoplasms of the pancreatic head.

The evolution and increasing use of parenchyma-sparing resection techniques—tumor enucleation, pancreatic middle segment resection, and duodenum-preserving pancreatic head resection—for benign tumors and cystic neoplasms have extended the surgeons’ skills and changed the indication to surgical treatment [12–14]. Patients suffering tumor-related symptoms or bearing asymptomatic pancreatic tumors are frequently aged under 50 years and have a long life expectancy. The use of parenchyma-sparing resection has significant advantages with respect to complications and late metabolic morbidity compared to classical pancreatic resections [15]. Duodenum-preserving pancreatic head resection (DPPHR) has the benefit of conservation of upper gastrointestinal tract (GI-tract) tissue and functions. With respect to benign or premalignant tumors of the pancreatic head, studies comparing open DPPHR with open PD have exhibited with high clinical evidence for lower early postoperative surgery-related complications following DPPHR [16], lower 90-day mortality [14], minimal metabolic morbidity [16], and maintenance of quality of life [17].

The following systematic review and meta-regression analysis was conducted to evaluate the contemporary state-of-the-art of laparoscopic DPPHR (L-DPPHR) for benign or premalignant neoplasms of the pancreatic head. Based on controlled clinical trials, L-DPPHRt and L-PD were compared with respect to intraoperative blood loss, operation (OP) time, need for blood transfusion, and the postoperative index complications. To evaluate the risk and benefits of laparoscopic total or partial DPPHR (L-DPPHRt or DPPHRp), the primary endpoints were the index metrics for early postoperative outcome [18]. Data about comparison of open DPPHR and open PD for benign, premalignant cystic neoplasms, and data of the advantages of DPPHR regarding early postoperative morbidity [14] and hospital mortality [16], endo- and exocrine pancreatic dysfunctions [15], and frequency of tumor recurrence [19] were recently published and not analyzed in this review.

## Materials and methods

### Search strategy

A comprehensive literature search was conducted in PubMed, Medline, Embase, and Cochrane Databases. For PubMed, a search with MeSH- and Emtree-terms was executed. A text word search for duodenum-sparing pancreatic head resection was performed. In a second step, the search was restricted to reports dealing with laparoscopic treatment of benign and premalignant neoplasms of the pancreatic head. Search terms were the following: laparoscopic duodenum-preserving pancreatic head resection—minimally invasive duodenum-preserving pancreatic head resection—parenchyma-sparing minimally invasive surgery for benign, premalignant pancreatic head tumors, and laparoscopic parenchyma-sparing resection for cystic neoplasms of the pancreatic head and laparoscopic PD for cystic neoplasms and PNETs.

Reports focusing on pancreatic head cancer and on inflammatory tumors of chronic pancreatitis were excluded. Publications about laparoscopic treatment of cystic neoplasms intraductal papillary mucinous neoplasm (IPMN), mucinous cystic neoplasm (MCN), solid pseudopapillary neoplasm (SPN), serous cystic neoplasm (SCN), and PNET and periampullary tumors were included in the search. The following criteria were separately listed: criteria for indication to surgery, preoperative and final histologic diagnosis, intraoperative data: OP time, blood loss, need for blood transfusion and tumor size, and the surgical index metrics for postoperative complications after L-DPPHR and L-PD.

**Fig. 1:**
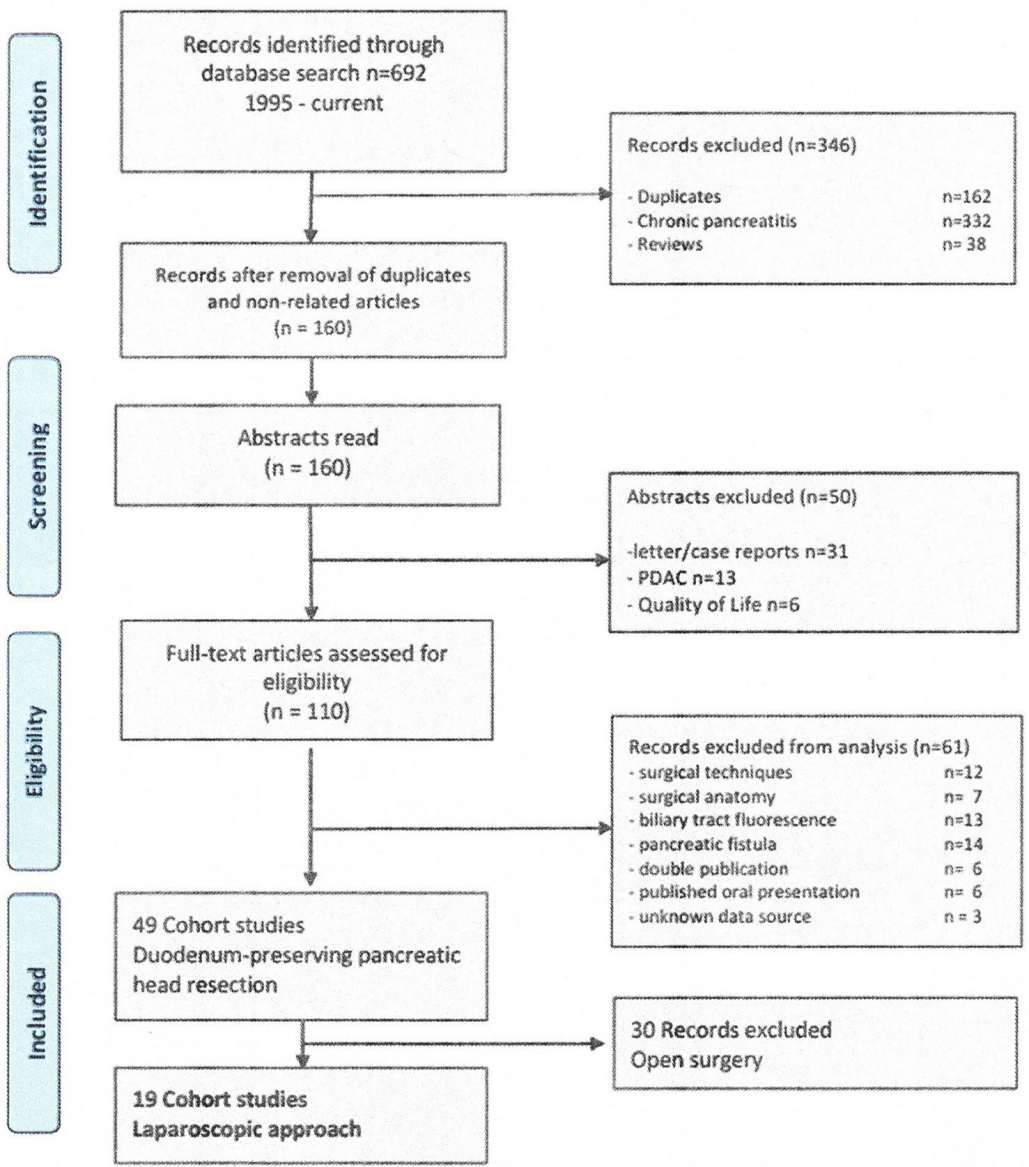
PRISMA flow diagram on the selection process of studies

Search results after identification of relevant publications including abstracts and full papers are presented in Fig. 1. Excluded were case reports, narrative case series, studies assessing pancreatic exo- and endocrine functions, and studies predominantly based on malignant tumors and studies with highly incomplete data sets. Two authors of the included publications were contacted to clarify final diagnosis [20, 21], missing early postoperative data, and the follow-up protocol (Table 1) [20–38].

**Table 1:** Baseline data and quality assessment of 459 patients undergoing L-DPPHR for benign tumors and premalignant neoplasms of the pancreatic head

Author/Ref	Publ. year	Study period	No. of pats. study *N*	Age years mean	Gender M/F	Type of DPPHR p/t/+ SD	Type of cohort study	Quality assessment	
								Oxford evid	NOS
Liang [22]	2024	2019–2021	10	35.2 ± 7.4	3/7	2/8/0^a^	Retrospective-controlled	2c	8
Zuo [23]	2024	2021–2023	43	55	23/20	0/43/0^a^	Prospective-controlled	2a	9
Wu [24]	2023	2020–2021	31	52	16/15	0/31/0^a^	Retrospective	2c	9
Liu [25]	2023	2016–2023	64	41.4 ± 15.7	40/24	0/64/0^a^	Retrospective	3a	9
Xia [26]	2023	2019–2022	31	48.4 ± 15.8	23/8	0/31/0^a^	Retrospective-controlled	2b	9
Zu [27]	2023	2016–2022	20	49	8/12	0/20/0^a^	Retrospective	3a	8
Huang [28]	2023	2015–2022	30	50.5	11/19	28/2/0^a^	Retrospective	3a	8
Zou [29]	2023	2014–2022	27	33.6 ± 10.3	2/25	0/27/0^a^	Retrospective-controlled	2a	9
Liu [30]	2022	2014–2020	12	58.5	9/3	0/12/0^a^	Retrospective-controlled^b^	3a	9
Xu [31]	2022	2021–2022	20	48.3	5/15	0/20/ 0^a^	Prospective	2c	8
Lu [32]	2022	2015–2021	25	46.7	8/17	24/1/0^a^	Prospective	2b	9
Zhou [33]	2022	4–12/2020	30	39.4	14/16	0/30/0^a^	Prospective	2b	9
Cai [34]	2021	2–11/2019	24	43	9/15	0/23/1^a^	Prospective	2b	9
Hong [35]	2020	2016–2019	22	46.7 ± 16	6/16	5/17 /0^a^	Prospective	2c	8
Chen [36]	2020	2016–2019	15	54.7 ± 13.9	4/11	0/15/0^a^	Retrospective-controlled^b^	2c	8
Cao [37]	2019	2016–2017	12	37.3	2/10	0/12/0^a^	Prospective	2c	8
Jiang [20]	2018	2016–2016	34	47 ± 14.7	8/25	0/34/0^c^	Retrospective-controlled^b^	2b	9
Thomas [38]	2015	2008–2013	5	64	ND	5/0/0^a^	Prospective-controlled^b^	2c	8
Peng [21]	2012	2010	4	42.3	1/3	0/4/0^c^	Prospective	3b	7

^a^Laparascopic DPPHR, partial,total, +SD

^b^Type of control group: Whipple or PPPD

^c^Study including patients with robotic assisted DPPHR

### Evaluation of methodological quality of studies

Nineteen cohort studies were included meeting the criteria of the search protocol. Assessment of the methods and results was performed using the Critical Appraisal Skills Program of the Oxford Centre of Evidence-Based Medicine [39]. Each cohort study was evaluated separately for the level of evidence. Selection criteria, measure bias, and applicability were assessed [40]. To identify the quality of the controlled prospective and retrospective cohort studies, the Newcastle–Ottawa Scale was applied, ensuring an objective evaluation of the most basic quality aspects of the non-randomized cohort studies with regard to selection criteria: case definition, representativeness of cases, selection of controls, comparability of study groups, and assessment of outcome variables by implementation of the internationally confirmed metrics of surgical results [41]. Cohort studies scoring 8 or 9 were considered acceptable or good level of evidence. Basically, the retrospective and prospective cohort studies reported prospectively collected surgical outcomes and clinicopathologic data except in six studies with retrospective-controlled group [20, 22, 26, 29, 30, 36]

### Modifications of DPPHR

**Fig. 2:**
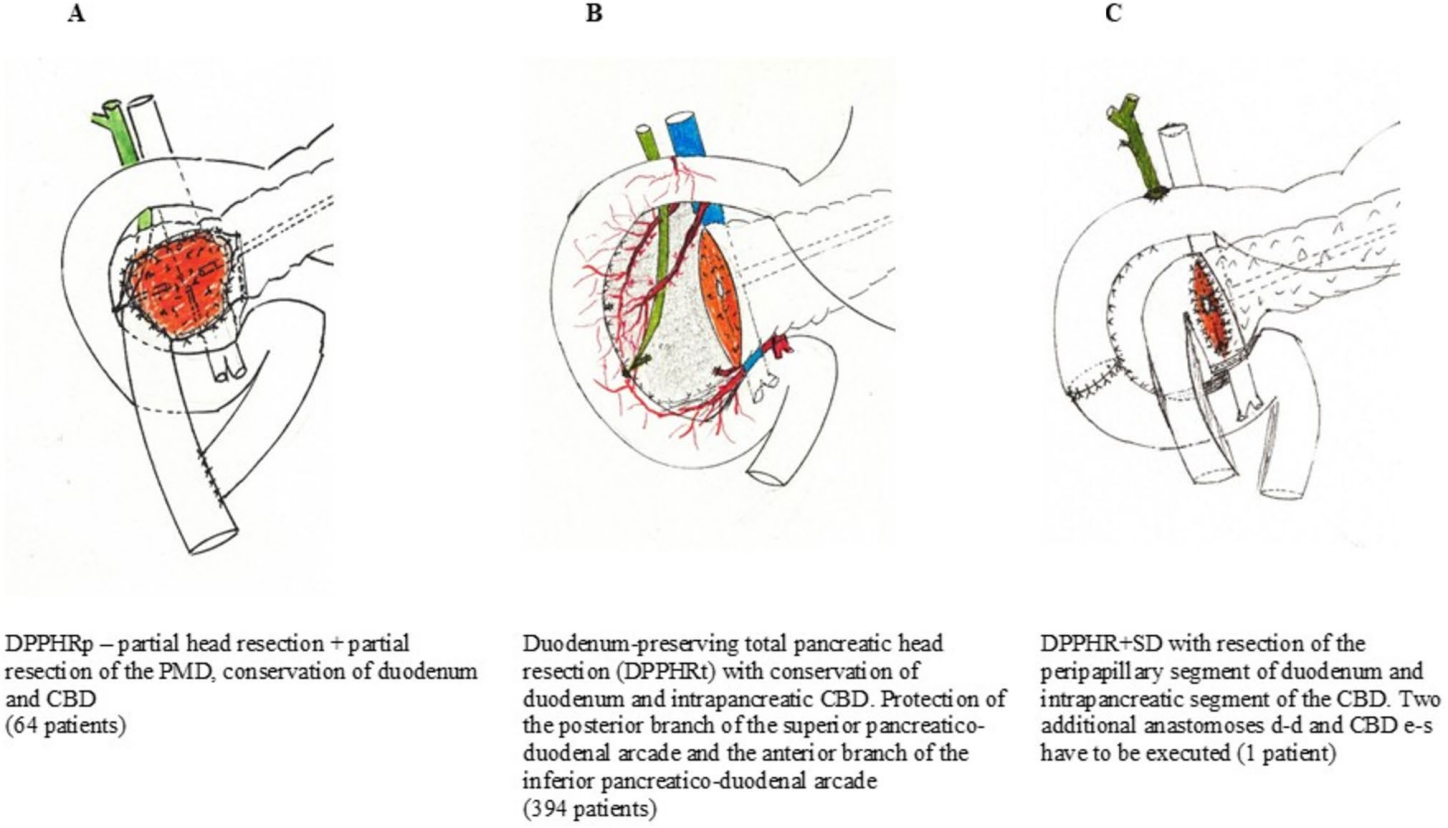
**A** DPPHRp—partial head resection; partial resection of the PMD, conservation of duodenum and CBD (64 patients) **B** duodenum-preserving total pancreatic head resection (L-DPPHRt) with conservation of duodenum and intrapancreatic CBD. Protection of the posterior branch of the superior pancreaticoduodenal arcade and the anterior branch of the inferior pancreaticoduodenal arcade (394 patients). **C** DPPHR + SD with resection of the peripapillary segment of duodenum and intrapancreatic segment of the CBD. Two additional anastomoses duodenum d-d and CBD e-s have to be executed (1 patient)

DPPHR is a parenchyma-sparing surgical technique comprising three minimally different types of local pancreatic head resection sparing the duodenum and common bile duct (CBD). Tumor size, tumor location in the pancreatic head, and proposed biological nature of the neoplasm determine the use of partial or total pancreatic head resection. With a few exceptions, partial pancreatic head resection preserves the duodenum and the CBD. DPPHRt requires resection of the pancreatic head, including the processus uncinatus, but preserves the duodenum, first jejunal loop, intrapancreatic CBD, and the neck of the pancreas (Fig. 2). Seven studies in the DPPHRt group applied near-total pancreatic head resection sparing some pancreatic tissue adherent to the intrapancreatic CBD and/or the peripapillary duodenum or of the suprapapillary pancreatic groove between CBD and duodenum. For large tumors, involving macroscopically the peripapillary duodenum or tumors of the periampullary region, a total pancreatic head resection with segment resection of the peripapillary duodenum and resection of the intrapapillary segment of the CBD was applied (DPPHR + SD). The modification of DPPHR + SD includes resection of the peripapillary duodenum and CBD but preserves about 90% of the duodenum (Fig. 2C). We have published the technical details of partial and total open pancreatic head resection for cystic neoplasms several times within the last 20 years [42, 43]. Figure 2A–C displays the procedural steps of the three modifications. Of note, in seven cohort studies, incomplete total pancreatic head resection was finally applied. By dissecting the intrapancreatic CBD and suprapapillary duodenum, some pancreatic tissue is left in situ intending to protect the blood supply of the peripapillary tissues [20, 21, 26, 28, 29, 31, 34]. After completion of the pancreatic head resection, the reconstruction of the GI tract is performed predominantly using the upper jejunal loop for pancreaticojejunostomosis (e-s anastomosis). A pancreatico-gastrostomosis (e-s) was executed in patients of four studies [25, 32, 37, 38]. DPPHR + SD requires three anastomoses: duodenostomy (e-e), CBD-duodenostomy (e-s), and pancreaticojejunostomosis (e-s).

### Data extraction process

Nineteen cohort studies were selectively evaluated according to a list of prespecified selection criteria. Data extraction for each study was conducted independently by two authors (HGB, BP). Each cohort study was re-evaluated five times to clarify differences of the reviewer. To assess the intraoperative and early postoperative outcome criteria, the newest surgical outcome metrics were applied based on recently published guidelines. Reinterventions were listed when surgery-related serious complications were managed by endovascular, radiologic, endoscopic, laparoscopic, or transhepatic methods to treat post-pancreatectomy bleeding, gastrointestinal bleeding, ischemic lesion of the peripapillary duodenal- and/or CBD sparing reoperation [44]. The presence of each criterion is listed in relation to the total number of patients. The variance in denominators of patients in the tables reflects the lack of data of the specific criteria and was consequently not included in the respective statistical calculations. The indications to L-DPPHR are listed as cumulative groups for surgery due to tumor caused symptoms based on main findings of imaging tools and randomly detected asymptomatic tumors (Table 2).

**Table 2:** Laparoscopic DPPHR in 459 patients for benign tumors, precancerous cystic neoplasms, premalignant neuroendocrine neoplasms, and other tumors of the pancreatic head: indication and type of L-DPPHR

Laparoscop./robotic-assist DPPHR pats	Public period	Patients* n*/*N*	Indication to surgery^d^			Type of L-DPPHR			Conversion^e^ L-DPPHRt to open DPPHRt or to PD pats	TM size cm	Follow-up time^f^ months
			Clinical symptoms	Imaging MRCP/CT/EUS	Randomly detected	L-DPPHRp (partial) pats	DPPHRt (total) pats	DPPHR + SD (+ segm. D/CBD) pats			
459^a^	2012–2024	**459**/1063^b^ 43.17%	**212** 46.18%	**132** 28.75%	**115** 25.05%	**64** 13.94%	**394** 85.83%	**1**^c^	**9** of 459 1.96%	3.66 ± 1.4	26.70 ± 11.50

Bold indicates the number of patients of the respective subgroup /to the index group

Robotic-assisted DPPHRt patients: 44/459 (9.58%)

^a^Lap./Robotic DPPHR—19 cohort studies

^b^Total of the Review Group 1063 patients after DPPHR [14]

^c^DPPHRt + SD—total pancreatic head + peripapillary segment resection of duodenum and CBD

^d^Dominant indicators to surgery: Clinical symptoms: upper abdominal pain, abdominal complaints/discomfort, attack of pancreatitis, jaundice; MRT/CT/EUS guideline-based: tumor growth, nodula, TM size ≥ 3 cm, PMD ≥ 9 mm, worrisome feature, suspected to be IPMN, MCN, SPN, CA 19–9 increase

^e^Conversion: 7 pats. L-DPPHRt → open DPPHRt; 1 pat. L-DPPHRt → open PD; 1 pat. L-DPPHRt one incision + 1 port → L-DPPHRt five port (21)

^f^13 cohort studies reported follow-up time

### Statistical analysis

All analyses were conducted using R for statistical computing (version 4.5.0., www.r-project.org, package meta). Continuous variables were expressed as mean and standard deviation (SD), and categorical variables were presented as absolute frequencies and percentages. Explorative statistical testing of L-DPPHRt versus L-PD (Tables 3, 4) was performed using the Welch-independent *t*-test, chi-square- or Fisher’s-exact-test, depending on the scale level of the variables to be tested. Statistical significance was set at *p* < 0.05.

The confirmatory meta-analysis for the two continuously scaled outcome parameters of primary interest, OP time and estimated blood loss, was conducted using the standardized mean difference (SMD). These confirmatory analyses, in contrast to the explorative testing approach in Table 4, used meta-analytic techniques (using an inverse variance weighting approach) in order to estimate the overall effect size [44]. In case of pronounced study heterogeneity (*I*^2^ > 50%), a random effects model has been applied to the data.

The meta-regression analysis was conducted based on a linear model including the intra- and postoperative outcome criteria of the group of L-DPPHR compared to L-PD [40]. For the analysis, the general health criteria age, male rate, body mass index (BMI), ASA-classification I and II, rate of chronic pancreatitis, rate of PNET, tumor size, history of previous abdominal surgeries, frequency of pancreatico-gastrostomosis, and DPPHR partial rate were used. The data were listed separately as supplemental material (Table S1, electronic supplementary material).

The individual follow-up times of each patient were not available in six reports [20, 23, 24, 34–36]. Therefore, the reported mean or median follow-up times of the respective study group were taken for the calculations. The overall follow-up response was 91.77% in the follow-up time of 26.70 months (mean). Follow-up times up to 90 postoperative days were taken as ‘no data’ (ND) for follow-up.

## Results

### Assessment of methodological quality of cohort studies

This systematic review was based on the results of 19 cohort studies (Table 1) to evaluate possible advantages of the use of L-DPPHR. Results of five retrospective-controlled cohort studies reporting data of 129 patients who underwent L-DPPHRt were compared to 205 patients who underwent L-PD. Applying the quality assessment of the Critical Appraisal Skills Program of the Oxford Centre for Evidence-Based Medicine revealed 14 studies with an evidence level 2a-c and five studies with an evidence level 3a-c [39, 40]. The Newcastle–Ottawa Scale (NOS) was additionally used to assess the quality of the prospective and retrospective cohort studies, ensuring an objective evaluation of the most basic quality aspects of non-randomized cohort trials; 18 studies displayed a score of ≥ 8, while one scored 7. The mean NOS score of the total group was 8.5, which indicates an acceptable to good quality of the included studies. A NOS score of 9 indicated a good study and a score of 7 a satisfactory quality [41].

### Baseline data

The baseline data of the 19 cohort studies comprising 459 patients (review group) are presented in Table 1. The 19 studies were published between 2012 and 2024. 18 of 19 cohort studies were published by Chinese groups, 1 from the US. In the review group, the mean age of patients was 47.0 years (SD ± 7.90) (Table 1). The gender relation M/F was 1 to 1.35. In the subgroup of meta-analysis comparing L-DPPHRt with L-PD, the mean age was 48.76 years and 55.97 years, respectively. The gender relation M/F was 1: 2.1 and 1: 0.8, respectively (Tables 4, S1). The gender relations M/F in the subgroups were 1: 1.3 and 1: 1.27, respectively.

### Final histopathology

Of 459 patients 367 with premalignant, cystic neoplasms underwent DPPHR (79.95%). Of them, IPMN (123 pats.) was the most frequent indication to parenchyma-sparing resection (33.51%). L-DPPHRt was preferentially applied for branch duct- (BD-) IPMN (Table 3). In two of the IPMN, the final histopathology revealed the stage of microcarcinoma, classified as pancreatic ductal adenocarcinoma (PDAC) stage 1A. SPN and SCA were found in one fourth of cystic neoplasms each. With regard to PNETs, DPPHRt was predominantly applied for benign, non-functional neoplasms (Table 3).

**Table 3:** Final histology of 459 patients after L-DPPHR of 367 patients with cystic precancerous neoplasms and in 59 patients premalignant neuroendocrine neoplasms of the pancreatic head

Total pats *N*	Cystic neoplasms							PNET		Other TM^c^	TM size
	IPMN^a^ (pats.)				MCN^b^ pats	SPN pats	SCA pats	Total pats	*nf*/*f* G1/2 pats	Pats	cm/mean
	Total	MD-^d^	BD-^d^	Micro- carcinoma							
459	**123** 33.51%	8/56	21/56	2	**44** 9.58%	**98** 21.35%	**102** 22.22%	**59** 12 ± 85%	48/11	**33** 7.18%	3.66 ± 1.4

Bold indicates the number of patients of the respective subgroup /to the index group

^a^Includes 2 patients with cancer arising in association with IPMN

^b^Includes 1 patient with MCN + severe dysplasia

^c^Comprises 30 patients with chronic pancreatitis and inflammatory tumor of the head and 3 patients with other cystic lesions

^d^8 of 19 studies reported differentiation of MD- and BD-IPMN

### L-DPPHRt versus L-PD

**Fig. 3:**
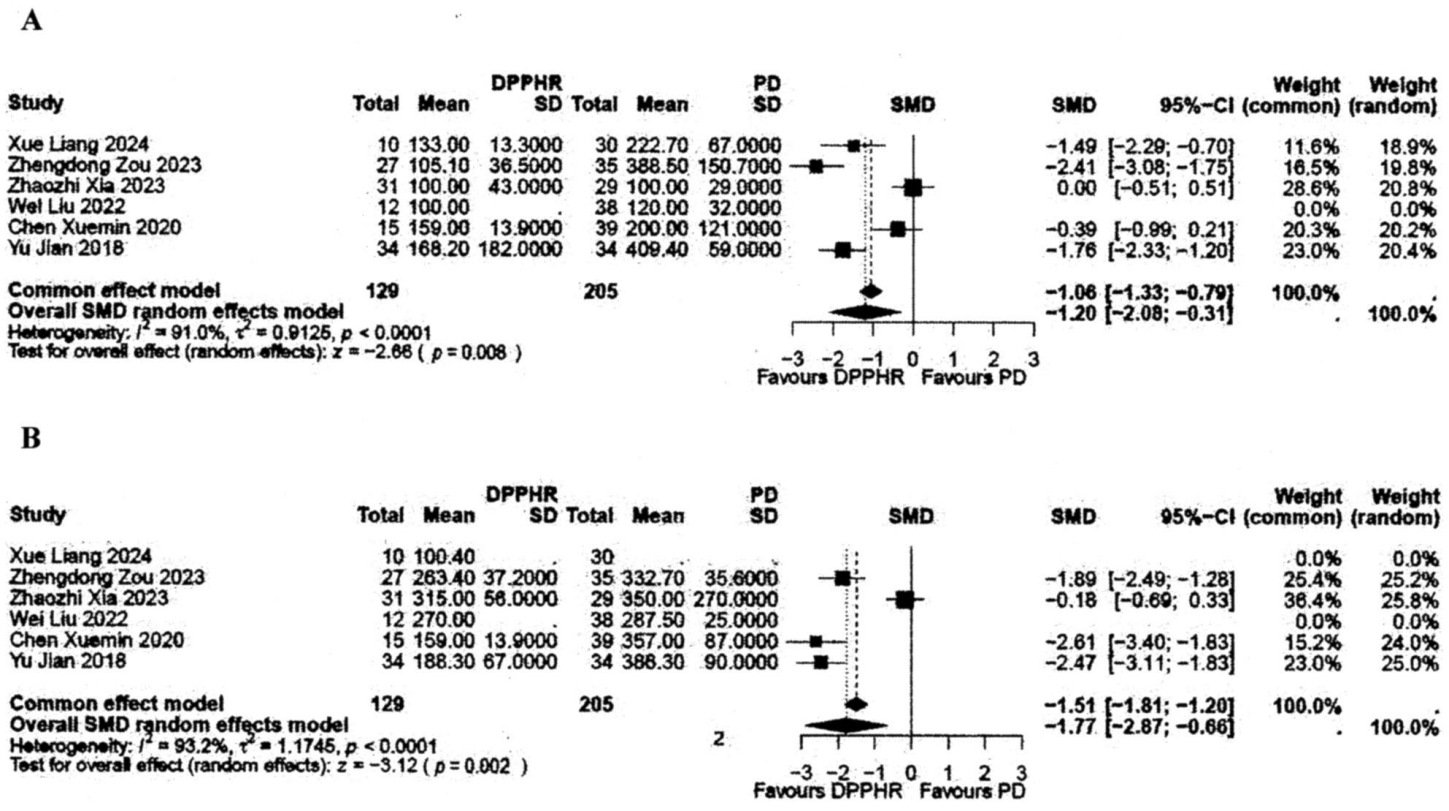
**A** + **B** Forest plots comparing L-DPPHRt (129 patients) with L-PD (205 patients) OP time (**A**) and estimated blood loss (**B**)

**Fig. 4:**
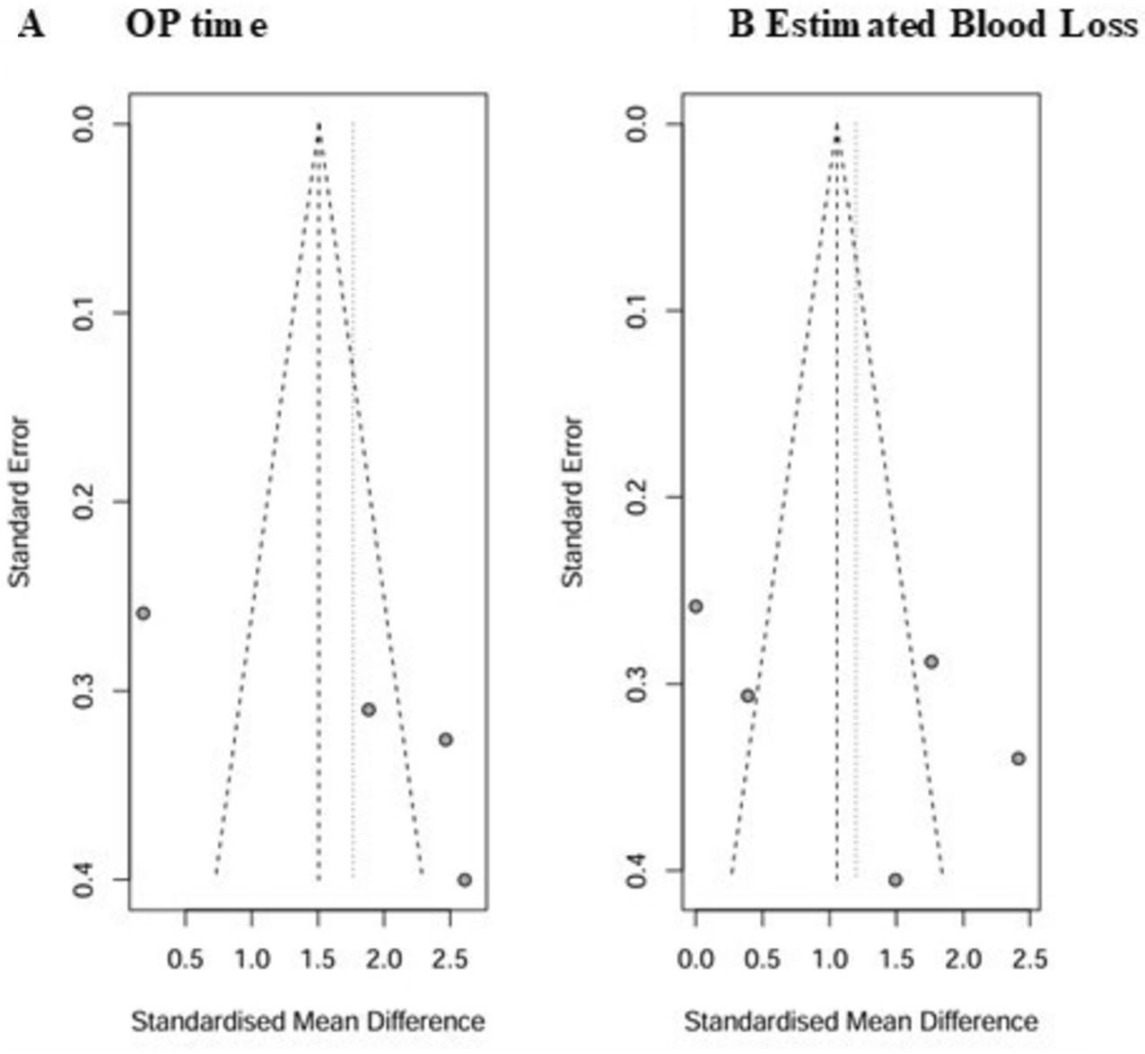
**A** + **B** Funnel plots comparing OP time and estimated blood loss following L-DPPHRt with L-PD

Six retrospective-controlled cohort studies reported results of early postoperative outcome comparing 129 patients, who underwent L-DPPHRt with preservation of duodenum and intrapancreatic CBD to L-PD in 205 patients. Laparoscopic Whipple procedure was performed in 117 patients and PPPD in 88 (Table 4). In the subgroup of laparoscopic comparison, tumor size was larger in the L-PD group. OP time was shorter, and the estimated intraoperative blood loss was lower in the L-DPPHRt group: 238.68 min. vs. 342.7 min. and 127.55 vs. 240.1 ml, respectively (Table 4). Both results demonstrated significant differences according to the meta-analysis (Fig. 3A/B). The funnel plot analysis revealed no indication for publication bias (Fig. 4A/B). Postoperative length of hospital stay (pLHS), frequencies of reintervention, and reoperation were clearly lower after L-DPPHRt, but not statistically significant compared to the results after L-PD. 90-day mortality after L-DPPHRt was one of 129 patients and one of 205 patients after L-PD (Table 4).

**Table 4:** Early postoperative outcome of laparoscopic DPPHRt (L-DPPHRt) versus laparoscopic PD (L-PD) for benign, premalignant neoplasms of the pancreatic head results of six retrospective, controlled cohort studies: [20, 22, 26, 29, 30, 36]

	Type of resection		*P* value
	L-DPPHRt^a^	L-PD^b^	
Patients (*n*)	129	205	
Study period	2017–2024	2017–2023	
Tumor size cm (mean)	**3.66** ± 0.97	**3.84** ± 1.49	NS^d^
OP time min. (mean)	**238.68** ± 80.31	**342.7** ± 36.42	*P* = 0.008^g^
Estimated blood loss ml (mean)	**127.55** ± 30.64	**240.1** ± 131.64	*P* = 0.002^g^
Blood transfusion *n*/*N* (%)	**6/129** 4.65%	**10/205** 4.87%	NS^e^
Reintervention *n*/*N* (%)	**9/129** 6.97%	**26/205** 12.68%	NS^e^
Re-operation *n*/*N* (%)	**3/129** 2.32%	**9/205** 4.39%	NS^f^
pLHS ^c^ days (mean)	14.9 ± 5.08	17.22 ± 3.32	NS^d^
Re-hospitalization *n*/*N* (%)	**3/89** 3.37%	**4/140** 2.85%	NS^f^
90-day mortality *n*/*N* (%)	**1/129** 0.77%	**1/205** 0.48%	NS^f^

Bold indicates the number of patients of the respective subgroup /to the index group

*NS* no significance

^a^L-DPPHRt: total pancreatic head resection with conservation of duodenum and CBD: 127 pats, L-DPPHRp: 2 pats

^b^L-PD: Whipple-OP 117 patients, PPPD 88 patients

^c^pLHS—Length of postoperative hospital stay

^d^Welch-independent t-test

^e^Pearson’s chi-square-test with Yate’s-continuity-correction

^f^Fisher-exact -test

^g^Forest plot

### Postoperative morbidity

The overall morbidity rate following L-DPPHRt for benign pancreatic head tumors was 43% and 39% following L-PD. The frequencies of pancreatic fistula POPF B/C [45] were 18.08%. Based on the reported surgical technique of L-DPPHRt, we identified seven studies with incomplete pancreatic head resection and 12 studies with complete pancreatic head resection. POPF B/C was observed in 14.06% in the complete and in 23.95% in the incomplete subgroup (Table 5). Biliary fistula A-C [46], including intraoperatively observed micro leaks of bile, was observed in 7.62% of patients. CBD stenosis in the pre-papillary segment was observed in six patients in the follow-up time of up to one year. Ischemic lesion of the peripapillary duodenum and the CBD occurred in two patients and required in one of 459 patients conversion to open DPPHRt [28]. A clinically relevant delay of gastric emptying (DGE) was observed in 5% of patients (Table 6). Reintervention and reoperation were of an acceptable low level with 7.62% and 2.47%, respectively. After L-DPPHRt, in-hospital- and 90-day-mortality revealed low levels of 0.21% and 0.43%, respectively. (Table 6).

**Table 5:** POPF following complete versus incomplete L-DPPHRt—results of 423 patients

L-DPPHRt	Patients total *N*	POPF (*n*)				
		*A*–*C* *n*/*N*	*A*	*B*	*C*	*B* + *C*
Complete^a^	256	64/256 pats	28 pats 10.93%	32 pats 12.54%	4 pats 1.56%	36 pats 14.06%
Incomplete^b^	167	60/167 pats	20 pats 11.97%	37 pats 22.15%	3 pats 2.39%	40 pats 23.95%
	*P* values^c^	= 0.016	= 0.863	= 0.013	= 0.999	= 0.030

^a^[22–25, 27, 30, 32, 33, 35–38]

^b^[20, 21, 26, 28, 29, 31, 34]

^c^Chi-square test

**Table 6:** Laparoscopic DPPHR in 459 patients for benign tumors, precancerous cystic neoplasms, premalignant neuroendocrine tumors of the pancreatic head: early postoperative outcome

Laparoscop./robotic-assist DPPHR	POPF *B* + *C*^a^ *n*/*N*	Biliary Leak/Fistula *A*–*C* *n*/*N*	CBD^b^ Stenosis *n*/*N*	Ischemic lesion of D/CBD^c^ *n*/*N*	DGE *B* + *C* *n*/*N*	Re-interv.^d^ *n*/*N*	Re-OP *n*/*N*	pLHS days (mean)	90-Day Re-hosp. *n*/*N*	90-Day mortality *n*/*N*
459 pats	**83/459** pats. 18.08%	**35/459** pats 7.62%	**6/459** pats 1.30%	**2/459** pats 0.43%	**14/280** pats 5.00%	**36/459** pats. 7.84%	**9/364** pats. 2.47%	459 pats 14.24 days	**8/224** pats. 3.57%	**2/459** pats. 0.43%

Bold indicates the number of patients of the respective subgroup /to the index group

^a^POPF B: 73 pats., POPF C: 10 pats.; included seven cohort studies L-DPPHRt with incomplete pancreatic head resection [20, 21, 26, 28, 29, 31, 34]

^b^CBD stenosis in the late postoperative outcome ≤ 1 year follow-up included

^c^Lesion of peripapillary segment of duodenum and common bile duct [28] and peripapillary CBD [37]

^d^Reintervention 19 pats. biliary fistula and/or CBD stenosis; 3 pats. temporary CBD stenting; 10 pats. bleeding/PPH—angio-radiologic embolization [23]; 4 pats. SSI/abscess drainage

### Use of L-DPPHR

L-DPPHRt was the predominantly applied procedure in 394 of 459 patients. L-DPPHRp with preservation of the intrapancreatic CBD, but with opening or partial resection of the pancreatic main duct was performed in 64 patients (13.94%) (Table 2). For IPMN, DPPHRt with preservation of the duodenum and the CBD was used. L-DPPHRt was preferentially applied for SPN, mainly found in female patients under the age of 40 years, because of larger size of the neoplasms. The reconstruction after completion of total pancreatic head resection was performed by pancreatico-jejunostomy using a Roux-en-Y excluded jejunal loop; only four studies reported the pancreatico-gastrostomosis to establish GI-tract continuity [25, 32, 37, 38]. One study applied in five patients the reconstruction by e-e anastomosis of the pancreatic main duct and tissue after subtotal pancreatic head resection [35].

Laparoscopic access to the abdominal cavity was achieved by standard 5-port technique. One study reported L-DPPHRt in 43 patients by single incision plus 1-port access [23]. 48 of 400 patients (12%) experienced previous abdominal surgery. Pneumoperitoneum was attained with carbon dioxide. In 44 of 459 patients (9.58%), robotic-assisted DPPHR (R-DPPHR) was performed using the DaVinci II system for robotic-assisted surgery [20, 21, 27, 31]. One prospective study with retrospective control group compared R-DPPHRt with R-PD in 34 patients in each group in terms of early postoperative outcome [20].

### Oncologic outcome and risk of recurrence after L-DPPHRt

The concordance between the preoperative diagnosis of a benign pancreatic head tumor of 459 patients undergoing L-DPPHR with a final, histopathologic diagnosis of benign neoplasm was 99.34%. In the mean follow-up time of 26.70 months, anastomotic and/or recurrence in the remnant pancreas was not observed, but liver metastasis was reported in one patient after IPMN resection. Intraoperative frozen section control of the resection margin was performed in all 459 patients. Data about tumor recurrence were reported for 212 patients who were followed by close surveillance protocol. Tumor recurrence was observed in the mean follow-up time of 26.70 months in two of 212 patients (0.94%). One patient developed an IPMN neoplasm in the remnant pancreas, and another patient developed liver metastasis.

## Discussion

Decision-making for patients bearing a cystic neoplasm or PNET is based on incomplete knowledge of the biologic nature of neoplasms and burdened with substantial inaccuracies of imaging tools to objectify presence and stage of the malignant transformation process [47, 48]. The aim of surgical treatment of premalignant neoplasm is to cure the patient before developing advanced cancer. Parenchyma-sparing pancreatic head resection has significant advantages regarding early postoperative complications and late metabolic morbidity [4]. Surgeons need to resolve the difficult decision-making between averting unnecessary surgery and preventing cancer disease. 46% of patients underwent surgery following clinical symptoms caused by the tumor. Data from imaging tools contributed to decision-making for surgical treatment in 29%, for most patients in concordance with guideline criteria (Table 2). According to the guidelines, clinically silent MCN, SPN, non-functional (nf-) PNETs are primarily candidates for surveillance [48, 49]. For patients with main-duct (MD-) IPMN, surgery is recommended without delay. IPMN and MCN with proof of severe dysplasia should undergo surgical treatment by using local resection [19]. With respect to SPN of the pancreatic head, the decision to use DPPHR and the laparoscopic approach was based on the young age of patients, including children and adolescents [50]. Most patients with SCN do not need surgical treatment, unless the tumor causes symptoms or displays tumor growth > 4 cm. However, 26% of patients with pancreatic head tumor were accidentally diagnosed, mostly SCNs and nf-PNETs. For patients bearing an accidentally detected neoplasm in the pancreatic head and who develop signs for tumor progression without clinical symptoms, local, parenchyma-sparing resection should be used. Additional lymph node allocation around the neoplasm is an important surgical step of L-DPPHRt for staging of the IPMN, MCN, SPN, and nf- /f-PNETs. Minimal risk for severe surgery-related complications, minimal mortality, and maintenance of pancreatic and upper GI-tract functions are additional advantages of using a parenchyma-sparing and not the classical resection.

Comparing open DPPHR with open PD, data of increasing evidence exhibited significant decrease in surgery-related, early postoperative complications after DPPHR [16]. Comparing L-DPPHRt and L-PD, the meta-analysis displayed the advantages of the laparoscopic approach. L-DPPHRt was associated with significantly shorter OP time and lower estimated intraoperative blood loss, fewer reinterventions, reoperations, DGE, and shorter pLHS, confirming the benefits of minimally invasive surgery (Table 4).

The low hospital mortality of 0.21% following DPPHR corresponds to reduced tissue trauma due to conservation of duodenum (DU), gastric antrum, and CBD. However, pancreatic and biliary fistula of 18.08% and 7.62%, respectively, remain the critical complications even after L-DPPHR. Ten of 83 clinically relevant POPF are grade C fistula. Incompletely resected pancreatic tissue, suture techniques, knot strength, blood supply, duct size, and tissue consistence are the well-established risks relevant for development of POPF or break of anastomosis [51]. Pancreatic fistula POPF B/C are the main determinant of early postoperative outcome after DPPHR. Incomplete pancreatic tissue resection was associated with a significantly higher risk for pancreatic fistula when applying laparoscopic total pancreatic head resection. As shown in Table 5, complete pancreatic head resection was associated with POPF B/C in 14.06%, whereas incomplete pancreatic head resection exhibited POPF B/C in 23.95%. Near-total pancreatic head resection is associated with a significantly higher frequency of POPF B/C. L-DPPHRt should be performed with the main focus of complete pancreatic tissue resection, particularly removing the tissue in the pancreatic groove between common bile duct and suprapapillary duodenal wall segment (Table 5).

Duodenal wall repair is occasionally required when dissecting the pancreatic head with large tumor causing extended abutment to the duodenal wall. Multiple sutures of the seromuscular layers of DU or a seromuscular patch using the jejunal loop may be a safe step to complete L-DPPHRt [33, 51]. Surgical institutions applying L- DPPHR referred to in this analysis are high volume centers for pancreatic surgery. However, the use of minimally invasive surgery, including robotic-assisted surgery, is still in a learning period. Data about the slope of the learning curve are missing.

The surgical anatomy between intrapancreatic segment of the CBD, DU, and pancreaticoduodenal arteries is complex and contains variable structures [52, 53]. Laparoscopic dissection of the CBD and pancreaticoduodenal arcades has due to magnification of small vessels and micro-structures the advantage of diminished tissue trauma and reduced blood loss. L- DPPHRt, used in 86% of patients, was the preferred technique of the parenchyma-sparing resection. After transection of the pancreatic neck and rotation of the head, Chen et al. recommend the inferior CBD triangle for easier identification of the CBD for total pancreatic head resection [36]. Dissection of the intrapancreatic segment of the CBD requires careful preservation of the wall of the CBD and the vascular supply of the suprapapillary CBD and the peripapillary duodenum. To maintain the blood supply of the peripapillary duodenum and intrapancreatic CBD, conservation of the posterior-superior and the anterior-inferior branch of pancreaticoduodenal arcades is most important to avoid tissue ischemia [54, 55]. Angiographically, a small artery running to the intrapancreatic segment of the CBD is documented, originating from posterior branch of the superior posterior pancreaticoduodenal arcade (SPDA) as frequently present [56, 57]. Ligation of the anterior branch of SPDA does not increase the risk for tissue ischemia of the peripapillary CBD/DU. Only one of 504 patients of the Ulm group who underwent DPPHR for inflammatory tumor in chronic pancreatitis with routine ligation of the anterior branch of the SPDA developed an ischemic duodenal fistula [58]. The intrapancreatic segment of the CBD varies in length between 2 and 4 cm [53]. In about 15% of patients, the CBD is free of surrounding pancreatic tissue, and only in the pre-papillary segment pancreatic tissue is circumferentially adherent. The blood supply of the intrapancreatic segment of the CBD is additionally sustained by small connective arteries originating from the posterior branch of the SPDA and the anterior branch of the inferior anterior pancreaticoduodenal arcade (IADA) [56].

Microtrauma of the CBD wall during L-DPPHRt dissection leads to biliary leakage or fistula, depending on the size of the leak. This explains the frequency of 7.62% bile leaks and fistula. With regard to biliary fistula A-C, the laparoscopic approach displayed a higher risk for biliary fistula C. In the group of L-DPPHRt, in 35 of 459 patients (7.62%), a biliary fistula was observed in the early postoperative days (Table 6). Most of the bile fistulas reported after L-DPPHRt are short-term biochemical bile leaking episodes, due to mini-trauma of the CBD during pancreatic tissue resection around the bile duct wall. Biliary fistula, in the early postoperative days with decreasing fluid amount and decreasing bilirubin concentration, is fistula A/B and not a serious complication [45]. Bile fistula C leading to clinical signs of cholangitis should be managed by short-term bile duct stenting in combination with short-term antibiotics. To minimize the risk of development of biliary fistula following intraoperative bile leakage or postoperative bile in the drainage tube, temporary CBD stenting causes drying of the source in most cases. In the postoperative follow-up time, 6 of 459 patients developed biliary stenosis and cholangitis episodes caused by CBD narrowing with the consequence of reoperation for new biliary-intestinal anastomosis. Thirty-six patients required reinterventions for serious complications of the Clavien–Dindo level ≥ III/IV. Biliary fistula (19 patients), post-pancreatectomy hemorrhage (PPH) (18 patients), and SSI/intraabdominal abscess (9 patients) were the most frequent serious complications after L-DPPHRt. CT-guided reintervention, embolization of the bleeding source, or interventional drainage resolved the serious complications. Only nine patients with severe complications underwent reoperation following L-DPPHRt. Two patients developed ischemic leaks of the peripapillary CBD and peripapillary duodenum, leading consequently to conversion to open PD in one patient and to open DPPHRt, respectively [28] (Table 6). Sutures of the duodenal wall and/or CBD stent placement, intraoperatively or by reintervention, have solved the complications. Concluding these data, laparoscopic DPPHRt was associated with a minimal risk of postoperative biliary duct complications.

To prevent bile duct trauma, the application of indocyanine green (ICG) fluorescence imaging of the biliary system may have significant advantages [59, 60]. Huang et al. observed bile leakage during L-DPPHRt to be clearly more frequent in the non-ICG-group compared to the ICG-Group [28]. After removal of the specimen, additional fluorescence biliography by real-time ICG imaging fluorescence to detect small injuries of the CBD wall leads to a reduced risk of postoperative biliary fistula [32, 61].

L-DPPHRt has the advantage of diminished operative trauma and does not increase the risk of CBD stenosis in the follow-up of one year (Table 6). Hence, L-DPPHRt has the quality to become the standard treatment for benign premalignant cystic neoplasms and PNETs of the pancreatic head. However, the results of the L-DPPHRt group reflect a highly selected population for minimally invasive pancreatic surgery (Table S1).

Contraindications to use the laparoscopic approach for surgical treatment of cystic and neuroendocrine neoplasms in the preoperative setting are the presence of a tumor harboring an advanced cancer or tumor size leading to extended compression of the pre-papillary CBD. Tumor location in the uncinate process is not a limitation for use of duodenum-preserving pancreatic head resection. Open DPPHRt is recommended for large neoplasms of the histological type of MD-IPMN or SPN > 6 cm. In the intraoperative setting, the technical reasons for conversion from L-DPPHRt are predominantly reported for persisting bleeding and intraoperative signs of ischemic lesion of the CBD wall or ischemic appearance of the peripapillary duodenum. In nine of 459 patients (1.96%), conversion of L-DPPHRt to open DPPHRt was executed; in four of nine patients due to persistent bleeding from resection of the pancreatic head along the suprapapillary duodenal wall, and in two patients (0.43%) for ischemic lesion of the duodenal wall and CBD (Table 6). Only one patient received a conversion to open PD for the combined ischemic lesion of duodenal wall and peripapillary duodenum.

### Strengths and limitations

We presented data of 459 patients who underwent laparoscopic and robotic-assisted DPPHR for premalignant, cystic neoplasms, and neuroendocrine tumors with respect to perioperative and oncologic outcome. We were able to demonstrate that laparoscopic DPPHRt ensures low early perioperative complications and minimal 90-day mortality. L-DPPHRt transfers additional benefits compared to L-PD regarding OP time, intraoperative blood loss, low frequencies of biliary fistula, and CBD stenosis. Due to conservation of the duodenum, first jejunal loop, and CBD, the basic objectives of DPPHR are maintenance of endocrine and exocrine functions of the pancreas and upper GI-tract, averting persistent late metabolic morbidity. The data presented are based on 19 cohort studies. Two of them are prospective- and six retrospective-controlled trials. Retrospective studies are inherently susceptible to selection and information bias. The six retrospective-controlled trials weaken the clinical evidence of the meta-analysis. Due to retrospective data accumulation, the risk of bias is increased. The reporting institutions unanimously are high volume centers for pancreatic surgery, however, with respect to DPPHR in a learning curve of accumulating procedure-specific expertise. The small number of prospective-controlled trials and the relatively low number of patients in the cohort groups limit the evidence of the message. Applying the quality assessment according to the Oxford Criteria of Evidence and the Newcastle–Ottawa scale criteria, the analyzed cohort studies display acceptable to good quality.

A prospective-randomized controlled comparison of DPPHR and PD for benign pancreatic head tumors is difficult to conduct due to patients’ education about the evidence of the advantages of DPPHR compared to PD regarding low early postoperative complications and the absence of long-term metabolic morbidity following duodenum- and CBD-preserving pancreatic head resection. A prospective-randomized comparison of partial DPPHR and tumor enucleation is warranted for small cystic neoplasms below 2 cm and for non-functional neuroendocrine tumors of the pancreatic head.

## Supplementary Information

Below is the link to the electronic supplementary material.

Supplementary file1 (PDF 148 kb)

Supplementary file2 (DOCX 23 kb)
