# Supplementary material for: Laparoscopic duodenum-preserving pancreatic head resection in 459 patients for precancerous, cystic neoplasms, and neuroendocrine tumors. Perioperative outcome: systematic review and meta-analysis
Source: Surg Endosc. 2026 Feb 6;40(3):1852–65. doi: 10.1007/s00464-026-12585-z (PMC12971798; doi:10.1007/s00464-026-12585-z)
Supplement: Supplementary file 1 — Supplementary file1 (PDF 148 kb) [file 464_2026_12585_MOESM1_ESM.pdf]

Laparoscopic Duodenum-preserving Pancreatic Head Resection

TITLE: Laparoscopic Duodenum-preserving Pancreatic Head Resection in 459 Patients for Precancerous, Cystic Neoplasms, and Neuroendocrine Tumors. Systematic Review and Meta-analysis of Perioperative Outcome

Background:
With regard to laparoscopic approach, the objective arises whether standard multiorgan Whipple resection (PD) or parenchyma-sparing procedures (DPPHRt) are the most qualified surgical treatments for benign, premalignant neoplasms.

Methods:
Pubmed, Embase, Medline and Cochrane Libraries were searched for studies reporting results and late outcomes after laparoscopic DPPHRt (L-DPPHRt) and laparoscopic PD (L-PD) for benign tumors. Data of 19 cohort studies including 459 patients were assessed. Results of six controlled trials comprising 129 L-DPPHRt and 205 L-PD for benign neoplasms were compared.

Beger et al. *Surgical Endoscopy*
Visual Abstract

Laparoscopic Duodenum-preserving Pancreatic Head Resection

Results:

L-DPPHRt was performed for 123 IPMNs, 44 MCNs, 98 SPNs, 102 SCNs and 59 PNETs. 90-day mortality was 2 of 459 patients (0.43%). Pancreatic fistula B/C occurred in 83 patients (18.08%), biliary fistula in 35 patients (7.62 %). Incidence of POPF B+C following complete and incomplete L-DPPHRt was 36/256 pats. (14.06%) and 40/167 pats. (23.95%) (p=0.030), respectively. LHS was 14.24 days (mean). Laparoscopic total DPPHR unveiled very low risk of hospital mortality (1/459 pats.;0.21%), reoperation (9/364 pats.;2.47%), DGE (14/280 pats.;5.0%), CBD stenosis (2/459 pats.;0.43%); and ischemic lesion of CBD (2/459 pats.;0.43%). Comparing 129 L-DPPHRt with 205 L-PD patients revealed overall mean values of 129 min. vs. 343 min. for OP time and 128 ml vs. 240 ml for estimated blood loss. Meta analysis using standardized mean difference (SMD) demonstrated these differences to be significant (OP time: SMD -1.20, 95% CI-2.08 to 0.31; p=0.008; blood loss: SMD -1.77, 95% CI -2.87 to -0.66; p=0.002). L-DPPHRt was associated with better intraoperative and early postoperative performance.

Conclusions:

Laparoscopic DPPHR for cystic neoplasms and PNETs is a low-risk procedure leading to cure of patients. L-DPPHRt accomplishes the most appropriate goals for treatment of patients with benign, premalignant, cystic neoplasms and PNETs (>2 cm) of the pancreatic head

Beger et al. Surgical Endoscopy
Visual Abstract
