# Supplementary material for: Laparoscopic duodenum-preserving pancreatic head resection in 459 patients for precancerous, cystic neoplasms, and neuroendocrine tumors. Perioperative outcome: systematic review and meta-analysis
Source: Surg Endosc. 2026 Feb 6;40(3):1852–65. doi: 10.1007/s00464-026-12585-z (PMC12971798; doi:10.1007/s00464-026-12585-z)
Supplement: Supplementary file 2 — Supplementary file2 (DOCX 23 kb) [file 464_2026_12585_MOESM2_ESM.docx]

**Table S1
Meta-regression Analysis of Data of the Controlled Cohort Studies Comparing L-DPPHRt with L-PD for Intra- and Early Postoperative Outcome (Supplemental Electronic Material)**

Outcome „OP time“

| ***Predictor*** | ***Estimate (SE)*** | ***p-value*** |
| --- | --- | --- |
| group (L-DPPHR vs. L-PD) | 104.46 (39.11) | 0.026 |
| age | 3.15 (2.39) | 0.221 |
| male rate | 23.93 (103.70) | 0.823 |
| ASA | 1.35 (2.64) | 0.628 |
| BMI | 19.07 (17.16) | 0.317 |
| CP rate | -88.80 (704.29) | 0.902 |
| PNET rate | -89.32 (292.79) | 0.767 |
| tumor size | -17.49 (22.58) | 0.459 |
| pre OP rate | -179.27 (262.71) | 0.512 |
| PG rate | -83.83 (51.33) | 0.137 |

Outcome „Blood loss”

| ***Predictor*** | ***Estimate (SE)*** | ***p-value*** |
| --- | --- | --- |
| group (L-DPPHR vs. L-PD) | 112.54 (55.18) | 0.069 |
| age | -2.65 (3.07) | 0.408 |
| male rate | -199.33 (127.22) | 0.152 |
| ASA | 3.22 (4.34) | 0.486 |
| BMI | 1.00 (33.96) | 0.978 |
| CP rate | 18.39 (920.29) | 0.984 |
| PNET rate | 212.72 (360.58) | 0.568 |
| tumor size | 20.78 (31.43) | 0.525 |
| pre OP rate | -162.15 (323.28) | 0.627 |
| PG rate | 70.73 (72.45) | 0.352 |

Outcome „Blood transfusion”

| ***Predictor*** | ***Estimate (SE)*** | ***p-value*** |
| --- | --- | --- |
| group (L-DPPHR vs. L-PD) | 1.00 (1.21) | 0.428 |
| age | -0.004 (0.061) | 0.944 |
| male rate | -2.59 (2.61) | 0.347 |
| ASA | 0.08 (0.10) | 0.443 |
| BMI | 0.78 (0.71) | 0.318 |
| CP rate | -6.25 (17.43) | 0.728 |
| PNET rate | 10.25 (6.20) | 0.129 |
| tumor size | -0.49 (0.59) | 0.427 |
| pre OP rate | 3.25 (6.15) | 0.609 |
| PG rate | 1.56 (1.36) | 0.279 |
